# Metformin alleviates oxidative stress‐induced senescence of human lens epithelial cells via AMPK activation and autophagic flux restoration

**DOI:** 10.1111/jcmm.16797

**Published:** 2021-07-23

**Authors:** Mengmeng Chen, Chunmei Zhang, Nan Zhou, Xu Wang, Dongmei Su, Yanhua Qi

**Affiliations:** ^1^ Department of Ophthalmology The Second Affiliated Hospital of Harbin Medical University Harbin China; ^2^ Department of Ophthalmology Xixi Hospital of Hangzhou Hangzhou China; ^3^ Department of Genetics Health Department National Research Institute for Family Planning Beijing China

**Keywords:** adenosine monophosphate, age‐related cataract, autophagic flux, metformin, oxidative stress, senescence

## Abstract

Cataracts are the leading cause of blindness worldwide owing to the increasing proportion of elderly individuals in the population. The purpose of this study was to investigate whether metformin could alleviate the occurrence and development of age‐related cataract (ARC) and the underlying mechanism. In the present study, we established a senescence model induced by oxidative stress, which was confirmed by measuring β‐galactosidase activity, qRT‐PCR and Western blotting. In addition, we showed that metformin alleviated the oxidative stress‐induced senescence of HLE‐B3 cells via the activation of AMPK. Next, we provided evidence that oxidative stress impaired autophagic flux and induced lysosomal dysfunction. Subsequently, we found that metformin restored autophagic flux that had been impaired by oxidative stress by activating AMPK. Additionally, we found that metformin suppressed HLE‐B3 cell senescence by improving lysosomal function and inactivating mTOR. Furthermore, the inactivation of AMPK, impairment of autophagic flux and lysosomal dysfunction were observed in the human lens epithelium of ARC. In summary, our data suggest that the activation of AMPK may be a potential strategy for preventing ARC, and metformin may be an emerging candidate to alleviate the formation and development of ARC.

## 1 INTRODUCTION

Cataracts are characterized by cloudiness or opacification of the eye lens and are the leading cause of blindness worldwide.^1^ Ageing is a main cause of cataracts and is responsible for more than half of the cases of cataracts (referred to as age‐related cataract [ARC]).^1^, ^2^, ^3^ ARC ranks as the most common type of cataracts, also known as senile cataract.^1^, ^4^ With the ageing of the population, the prevalence of ARC is increasing each year, which not only seriously affects the health and quality of life of patients but also causes enormous economic and social burdens.^5^, ^6^ At present, the generally accepted treatment of ARC is surgical intervention.^7^ Surgical treatment not only brings certain economic pressure to the patient but is also affected by the age of the patient and surgical stress. Most patients will have obvious psychological disorders during the operation. In addition, postoperative complications such as infection, corneal astigmatism, corneal oedema, iris damage, ligament damage, glaucoma and posterior cataracts may occur. Some patients even need to undergo reoperation.^8^

Although surgery is the main treatment for ARC, the complications associated with surgery cannot be ignored. Therefore, it is urgent to explore the pathogenesis of ARC and identify target genes and effective drugs for the treatment of ARC. Numerous studies have demonstrated that genes and environmental factors, including ultraviolet rays, ionizing radiation, chemicals and DNA damage, contribute to ARC.^9^, ^10^ One of the mechanisms of the above‐mentioned interactions is that oxidative stress is triggered and further leads to the development of ARC.^10^, ^11^ In the past few years, research on anti‐ageing treatment has discovered many drugs that can prolong life, among which metformin (MET) is the most notable. It has been found that MET not only lowers blood sugar, prevents macrovascular and microvascular diseases, and improves hyperinsulinaemia and insulin resistance^12^ but also delays ageing, inhibits age‐related pathological changes and reduces oxidative stress damage.^13^, ^14^ If the mechanism of action of MET can be clearly explained, its clinical application will be expanded to treat certain age‐related diseases, including ARC, and increase life expectancy.

The majority of MET studies have demonstrated that MET can prolong the life span of mice and *C*. *elegans* and plays an important role in enhancing the health of these organisms. MET increases the average life span of *C*. *elegans* by 40%, prolongs the health of *C*. *elegans*, slows the accumulation of lipofuscin and prolongs young motor ability in a dose‐dependent manner.^15^ Similarly, at the age of 12 months, C57BL6 mice were fed 0.1% MET for 6 months. The average life span of C57BL6 mice was prolonged by 5.83%. The average life span of B6C3F1 mice was prolonged by 4.15% when the mice were fed in the same way and for the same time as C57BL6 mice.^16^ Furthermore, a 10‐year randomized clinical trial used MET to treat overweight/obese patients with type 2 diabetes and showed that long‐term use of MET is beneficial to human health and survival.^17^

Lens epithelial cells, a single layer of epithelial cells on the lens’ anterior surface, are the most metabolically active part of the lens. They provide basic materials and metabolic energy for the growth, differentiation and damage repair of the lens. The normal construction and function of lens epithelial cells is essential for the maintenance of the transparency and metabolic homeostasis of the entire lens.^18^ Previous studies have revealed that oxidative stress, especially H_2_O_2,_ could cause excessive accumulation of reactive oxygen species (ROS), resulting in dysfunction and irreversible damage of normal lens epithelial cells, contributing to the modification, denaturation, aggregation of lens proteins including enzyme and crystallins, initiating early cataract formation.^19^, ^20^ Therefore, the mechanisms of protecting the normal lens epithelial cells against oxidative stress are an ongoing focus in the field of ARC research.

In the present study, we clarified the role and specific mechanism of MET in our system. Our results indicate that (a) MET can delay the hydrogen peroxide‐induced senescence of lens epithelial cells by activating the AMPK pathway, (b) MET restores autophagic flux by activating the AMPK pathway, and (c) MET restores autophagic flux associated with the amelioration of lysosomal function and mammalian target of rapamycin (mTOR) inactivation. Our study provides a rationale for cellular senescence‐based therapeutics for the protection of the eye and for the treatment of ARC in the elderly population.

## 2 MATERIALS AND METHODS

### 2.1 Study participants and preparation of human anterior lens capsules

Sixty‐four patients aged 50–55 years with ARC participated in this study. Human lens epithelium specimens (approximately 5 mm in diameter) were collected during cataract surgery. Normal lens anterior capsule specimens with the adherent epithelium (approximately 5 mm in diameter) were donated by the patients and served as controls in the study.^21^ We declared that the study followed the tenets of the Declaration of Helsinki and was approved by the Ethics Committee of Harbin Medical University. All subjects in this study knew and understood the content and risk of the research and signed the informed consent form.

According to Lens Opacities Classification System III (LOCS III), patients whose lenses had a score of C2‐C3, N2‐N3, or P2‐P3, including eight patients in each ARC category, and eight age‐matched controls from donor eyes from the eye bank with a LOCS III score of ≤C1, ≤N1 or ≤P1 were enrolled.^22^

### 2.2 Haematoxylin and eosin (H&E)

Fresh anterior lens capsules (human) were immediately fixed with 4% paraformaldehyde at room temperature and embedded in paraffin. For histological examination, the tissues were cut into 4‐μm slices. The sections were deparaffinized with 100% xylene and then rehydrated with gradient alcohol (100% ethanol, 90% aqueous ethanol, 80% aqueous ethanol, 70% aqueous ethanol and distilled water). Then, the sections were stained with H&E (5 min and 2 min, respectively, at room temperature), and dehydrated. The morphological changes were observed under a microscope (Nikon, Eclipse).

### 2.3 Senescence‐associated β‐galactosidase (SA‐β‐Gal) assay

SA‐β‐Gal activity was measured by using an SA‐β‐Gal staining kit (Sigma, CS0030). According to the manufacturer's instructions, tissue sections and HLE‐B3 cells after a series of treatments were washed with phosphate‐buffered saline for 5 min (thrice) and then incubated in SA‐β‐gal staining solution (PH6.0) at 37℃ without CO_2_ for 24 h. The images were captured under a light microscope (Nikon, Eclipse). The percentage of SA‐β‐gal‐positive cells was estimated as the mean number of positive cells/the mean number of total cells.

### 2.4 Immunohistochemistry (IHC)

Paraffin sections were deparaffinized and hydrated through a xylene and graded alcohol series. The sections were rinsed with water, boiled in 0.1 M citric acid (pH 6.1) for 30 min, allowed to cool to room temperature and treated with 3% hydrogen peroxide/deionized water buffer to inhibit endogenous peroxidase. Then, the fixed capsules were blocked with foetal bovine serum (FBS) for 30 min at room temperature and incubated with anti‐p21, anti‐p53, anti‐phospho‐AMPKa (Thr172), anti‐phospho‐ACC (Ser79), anti‐LC3, anti‐SQSTM1/p62 and antibodies in PBS overnight at 4℃. The secondary antibody conjugated to horseradish peroxidase (Cell Signaling Technology, USA) was then applied for 1 h at 37℃. Immunoreactivity was detected using diaminobenzidine (DAB; Cell Signaling Technology), and then, coverslips were added with Permount mounting medium. Immunostained images were captured using a Nikon Eclipse microscope (Nikon, Eclipse).

### 2.5 Western blot analysis

Total protein was extracted from human lens epithelial tissue or cultured cells using RIPA lysis buffer with a protease inhibitor cocktail (Pierce), and a BCA kit (Thermo Scientific) was used to quantify the protein concentration. Forty micrograms of protein was loaded on an SDS‐PAGE gel and separated by electrophoresis, followed by blotting onto a PVDF membrane (Millipore). The target proteins were probed with the corresponding primary antibodies against P21, P53, AMPKa1, phospho‐AMPKa (Thr172), phospho‐ACC (Ser79), LC3, SQSTM1/p62, CTSB, phospho‐mTOR (Ser2448), phospho‐p70S6K under optimized conditions and then incubated with the secondary antibody. Immunological signals were visualized via the electrochemiluminescence method using an Immobile Western Chemiluminescence HRP substrate kit (Millipore).

### 2.6 Reagents

MET, hydroxychloroquine (HCQ) and rapamycin (RAPA) were purchased from Sigma‐Aldrich (CA); compound C (CC) was purchased from APExBIO. Antibodies against LC3 (12741), phospho‐ACC (Ser79) (3661), phospho‐mTOR (Ser2448) (5536), phospho‐p70S6K (Thr389) (9206) and p53 (2524) were purchased from Cell Signalling Technology. Antibodies against p21 (ab109520) p53(ab31333), phospho‐AMPKa (Thr172) (ab133448), AMPKa1 (ab32047), β‐actin (ab8226) and SQSTM1/p62 (3340–1) were obtained from Abcam.

### 2.7 Cell culture

The human lens epithelial cell line (HLE‐B3) was cultured in Dulbecco's modified Eagle's medium (DMEM, GIBCO) supplemented with 20% FBS, penicillin (100 U/ml) and streptomycin (100 mg/ml) in a humidified incubator with 5% CO_2_ at 37℃.

### 2.8 Quantitative real‐time polymerase chain reaction (qRT‐PCR)

Total RNA was isolated from cultured cells using TRIzol reagent (Invitrogen) and reverse transcribed using the PrimeScript RT reagent kit (Takara) according to the manufacturer's instructions. qRT‐PCR was performed using a SYBR Green Supermix kit (Takara), with β‐actin as an endogenous control. The primer sequences used for PCR are shown in Table S1

### 2.9 Statistical analysis

Student's t‐tests and ANOVA were used to calculate the statistical significance of the experimental data. The significance level was set as *p < 0.05 and **p < 0.01. Error bars denote SD.

## 3 RESULTS

### 3.1 Identification of senescent HLE cells in the human lens epithelium of ARCs and controls

**FIGURE 1:**
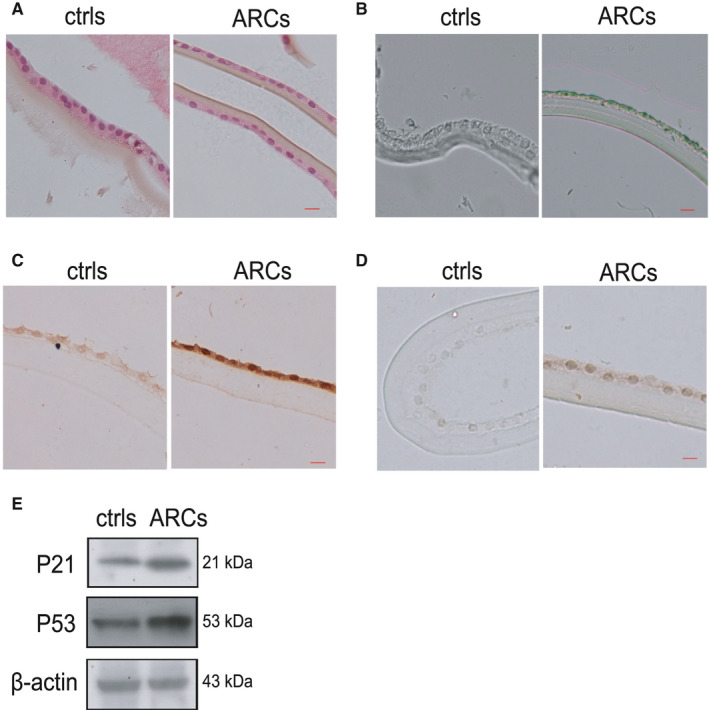
Identification of senescent HLE cells in the human lens epithelium of ARCs and controls (A). The human lens epithelium of in the controls (left) and ARCs (right) was analysed by H&E staining. (B). Representative images of SA‐β‐Gal staining of ARCs and the controls. (C). Representative images from IHC assays against P53 in the controls (left) and ARCs (right). (D). Representative images from IHC assays against P21 in the controls (left) and ARCs (right). (E). Western blot analysis of P53 and P21 in the controls and ARCs. The bar represents 50 um

To investigate the senescence of lens epithelial cells in the lens epithelium of ARCs and the controls, we performed a series of measurements. H&E and SA‐β‐Gal staining were used to detect morphological differences in lens epithelial cells in ARC and control samples. The results indicated that lens epithelial cells became flat and sparse in the ARC samples compared to the control samples (Figure 1A). Moreover, SA‐β‐Gal staining showed that senile lens epithelial cells were strongly positive (Figure 1B). The IHC results demonstrated significant differences in senescence‐related genes (P53, P21) between ARCs and the controls (Figure 1C,D). Western blot analysis confirmed the IHC results showing the expression of senescence‐related genes (P53, P21) in ARCs were significantly higher than those in the control samples (Figure 1E).

### 3.2 Oxidative stress‐induced senescence in HLE‐B3 cells in vitro

**FIGURE 2:**
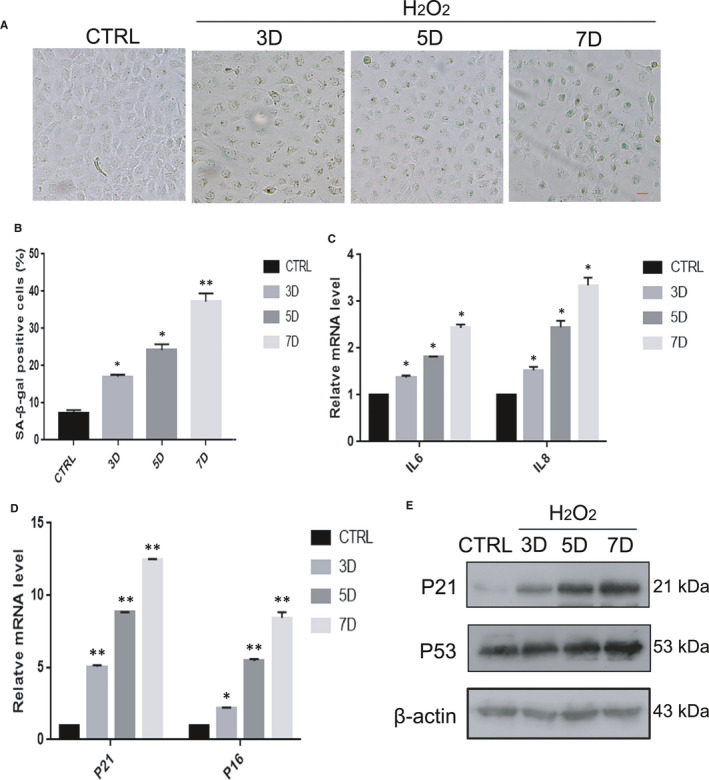
Oxidative stress‐induced senescence in HLE‐B3 cells in vitro. HLE‐B3 cells were treated with H_2_O_2_ for 3–7 days. CTRL means untreated cells. (A). Representative images of SA‐β‐Gal staining of the cells. (B). Percentages of SA‐β‐Gal‐positive cells. (C). Relative fold‐changes in the mRNA levels of the genes encoding IL6 and IL8, as determined by qRT‐PCR. (D). Relative fold‐changes in the mRNA levels of the genes encoding P53 and P21, as determined by qRT‐PCR. (E). Western blot analysis of P53, P21 and β‐actin in HLE‐B3 cells treated with H_2_O_2_ (150 μM) for 3–7 days. Data were shown as mean ± SD and are representative of 3 independent experiments. *p<0.05; **p < 0.01 compared to the control (CTRL). The bar represents 20 um

Exogenous H_2_O_2_ has been widely used as a potent inducer of cellular senescence, which is commonly referred to as oxidative stress‐induced senescence.^23^ To establish cellular senescence in response to oxidative stress in human lens epithelial cells, HLE‐B3 cells were treated with 150 µM H_2_O_2_ for 1 h and were then incubated in complete medium for adhesion culture for 3, 5, and 7 days. The results showed that H_2_O_2_ exposure induced the cell morphology to become large and flat. SA‐β‐Gal staining was also strongly positive in these cells (Figure 2A,B). These morphological changes were consistent with the increased expression of four other senescence‐associated genes (P53, P21, P16, IL6 and IL8) (Figure 2C,D,E). Furthermore, with prolonged exposure to H_2_O_2_, not only the proportion of senescent cells but also the mRNA and protein expression of senescence marker genes gradually increased. Therefore, treatment with 150 μM H_2_O_2_ for 7 days was chosen to induce oxidative stress in HLE‐B3 cells for subsequent experiments.

### 3.3 The AMPK pathway was inactivated in the human lens epithelium of ARCs and in HLE‐B3 cells with oxidative stress‐induced senescence

**FIGURE 3:**
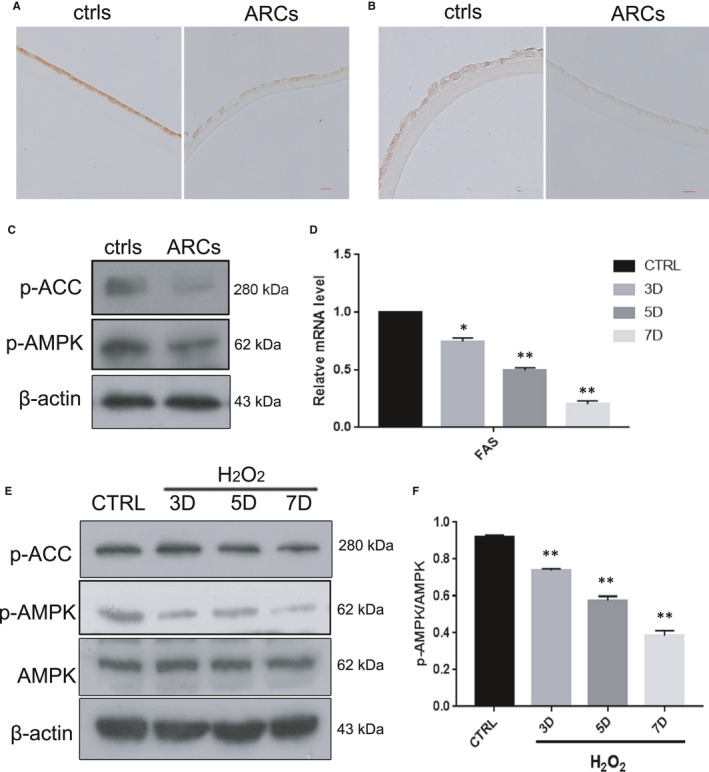
The AMPK pathway was inactivated in the human lens epithelium of ARCs and in HLE‐B3 cells with oxidative stress‐induced senescence (A). Representative images from IHC assays against p‐AMPKα (Thr172) in the controls (left) and ARCs (right). (B). Representative images from IHC assays against p‐ACC (ser79) in the controls (left) and ARCs (right). (C). Representative images from Western blot analysis of p‐AMPKα (Thr172), p‐ACC (ser79) and β‐actin in the controls (left) and ARCs (right). (D). Relative fold‐changes in the mRNA levels of the genes encoding FAS, as determined by qRT‐PCR. (E). Western blot analysis of AMPKα, p‐AMPKα (Thr172), p‐ACC (ser79) and β‐actin in HLE‐B3 cells treated with H_2_O_2_ (150 μM) for 3–7 days. (F). Relative fold‐changes in the protein levels of p‐AMPKα/AMPKα as described in E. Data were shown as mean ± SD and are representative of 3 independent experiments. *p < 0.05; **p < 0.01 compared to the control (CTRL). The bar represents 50 um

Accumulating evidence indicates that inactivation of the AMPK pathway is closely related to ageing.^24^, ^25^ Based on this theory, we measured the expression of AMPK pathway components in human lens epithelium and HLE‐B3 cells in vitro. The protein levels of phosphorylated AMPKα (Thr172) and phosphorylated ACC (Ser79) were markedly decreased in ARCs, while the total protein levels of AMPKα remained unchanged (Figure 3C). Analogously, IHC examination showed that the expression levels of the critical genes p‐AMPKα (Thr172) and p‐ACC (Ser79) were decreased in the anterior capsule of the lens in ARCs compared with the controls (Figure 3A,B). Next, we found that the mRNA expression of FAS, an important gene that is downstream of the AMPK pathway, was significantly decreased in oxidative stress‐induced HLE‐B3 cells (Figure 3D). Unsurprisingly, the protein levels of phosphorylated AMPKα (Thr172) and phosphorylated ACC (Ser79) were also significantly reduced in the senescent HLE‐B3 cells (Figure 3E). These results revealed that the AMPK pathway was inactivated in both the lens epithelium of ARCs and HLE‐B3 cells with oxidative stress‐induced senescence.

### 3.4 MET alleviated oxidative stress‐induced senescence in HLE‐B3 cells

**FIGURE 4:**
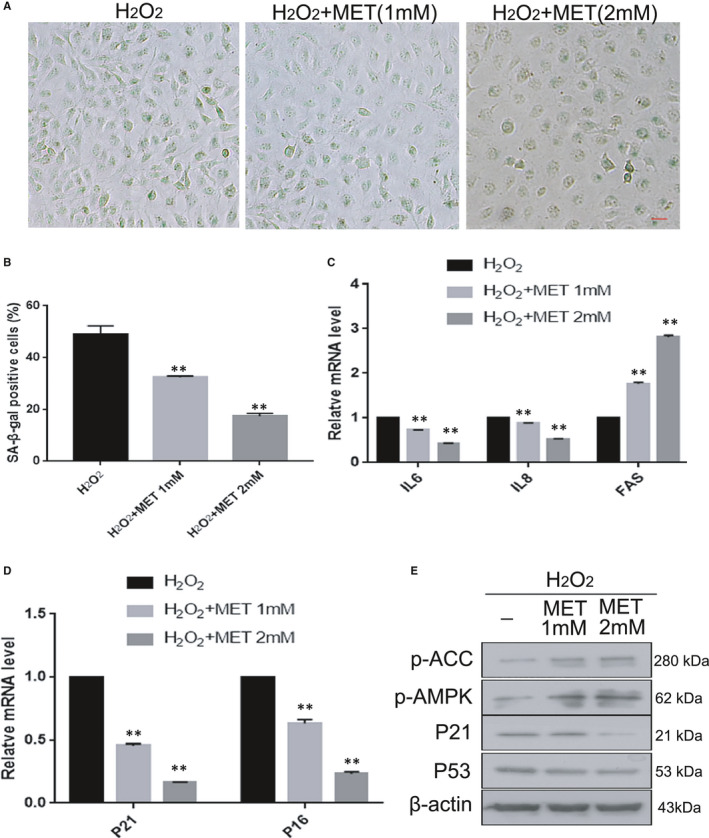
MET alleviated oxidative stress‐induced senescence in HLE‐B3 cells. HLE‐B3 cells were treated with MET at different concentrations. **(**A). Representative images of SA‐β‐Gal staining of the cells. (B). Percentages of SA‐β‐Gal‐positive cells. (C). Relative fold‐changes in the mRNA levels of the genes encoding IL6, IL8, FAS, as determined by qRT‐PCR. (D). Relative fold‐changes in the mRNA levels of the genes encoding P21 and P16, as determined by qRT‐PCR. (E). Western blot analysis of P53, P21, p‐AMPKα (Thr172), p‐ACC(ser79) and β‐actin in HLE‐B3 cells treated with MET at different concentrations. Data were shown as mean ± SD and are representative of three independent experiments. *p < 0.05; **p < 0.01 compared to the indicated sample. The bar represents 50 um

To evaluate the effects of the known AMPK pathway activator MET on oxidative stress‐induced senile lens epithelial cells, different concentrations of MET were added to the culture medium after H_2_O_2_ treatment. As shown in Figure 4A,B, the number of SA‐β‐gal‐positive cells significantly decreased in a robust concentration‐dependent manner. As expected, MET reduced the mRNA expression of senescence‐associated genes (P21, P16, IL6 and IL8) (Figure 4C,D). In addition, the protein levels of P21 and P53 were markedly decreased (Figure 4E). Thus, we selected 2 mM MET as the optimal concentration for our subsequent experiments. Moreover, in order to better illustrate the anti‐ageing mechanism of MET, we performed three experiments between control and metformin alone groups in HLE‐B3 cells: SA‐β‐Gal staining, qRT‐PCR and Western blot analysis. These data showed that there was no significant difference in the expression of senescence‐associated genes (P21, P16, P53) between control and MET alone groups (Figure S1). Therefore, MET may prevent the senescence of HLE‐B3 cells against oxidative stress.

### 3.5 MET prevented oxidative stress‐induced senescence in HLE‐B3 cells via AMPK activation

**FIGURE 5:**
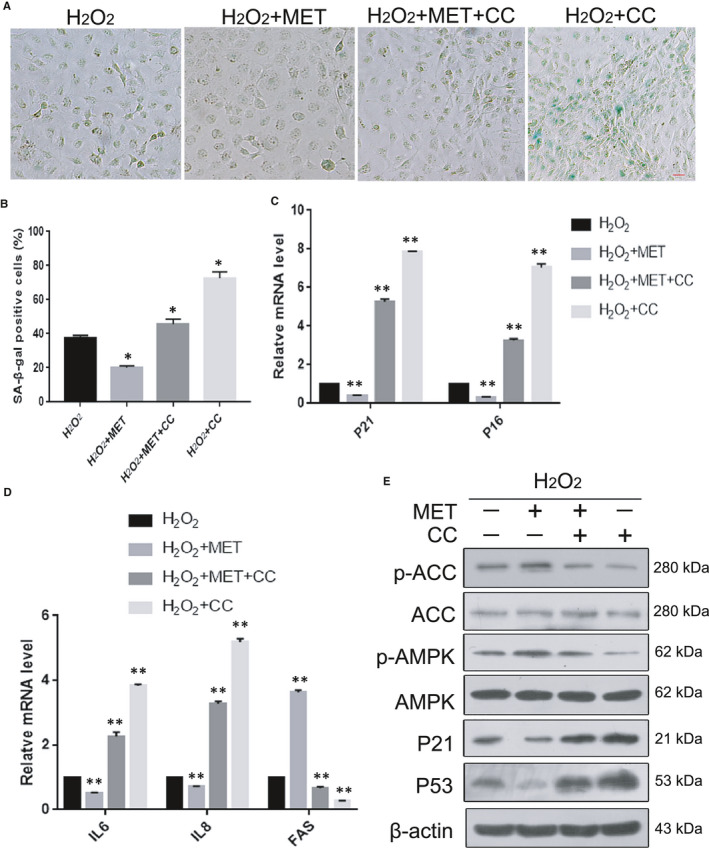
MET prevented oxidative stress‐induced senescence in HLE‐B3 cells via AMPK activation (A). Representative images of SA‐β‐Gal staining of the cells. (B). Percentages of SA‐β‐Gal positive cells. (C). Relative fold‐changes in the mRNA levels of the genes encoding P21 and P16, as determined by qRT‐PCR. (D). Relative fold‐changes in the mRNA levels of the genes encoding IL6, IL8, FAS, as determined by qRT‐PCR. (E). Western blot analysis of P53, P21, AMPKα, p‐AMPKα (Thr172), ACC, p‐ACC (ser79) and β‐actin in HLE‐B3 cells treated with H_2_O_2_ (150 μM), MET (2 mM) and CC (4 mM). Data were shown as mean ± SD and are representative of 3 independent experiments. *p < 0.05; **p < 0.01 compared to the indicated sample. The bar represents 20 um

MET is known to activate the AMPK pathway, which plays an important role in delaying senescence.^26^ The proportion of senescent cells was reduced when MET activated the AMPK pathway in HLE‐B3 cells with oxidative stress‐induced senescence. Treatment with CC, a known inhibitor of the AMPK pathway, markedly increased the number of SA‐β‐Gal‐positive cells. The combination of H_2_O_2_ and CC showed a stronger effect in stimulating senescence than that of H_2_O_2_ alone. However, when combined with MET, the CC‐mediated exacerbation of senescence was largely blunted, as indicated by the marked decrease in SA‐β‐Gal positivity (Figure 5A,B). Likewise, the protein expression of P53 and P21 confirmed the efficiencies of CC and MET treatments, as assessed by western blotting (Figure 5E). Next, we measured the mRNA expression level of FAS and the protein levels of phosphorylated AMPKα (Thr172) and phosphorylated ACC (Ser79) and further verified that MET alleviated CC‐induced inhibition of the AMPK pathway in our senescence system. These results indicated that MET prevents oxidative stress‐induced senescence in HLE‐B3 cells via AMPK activation.

### 3.6 Impairment of autophagic flux and lysosomal dysfunction in the human lens epithelium of ARCs and in HLE‐B3 cells with oxidative stress‐induced senescence

**FIGURE 6:**
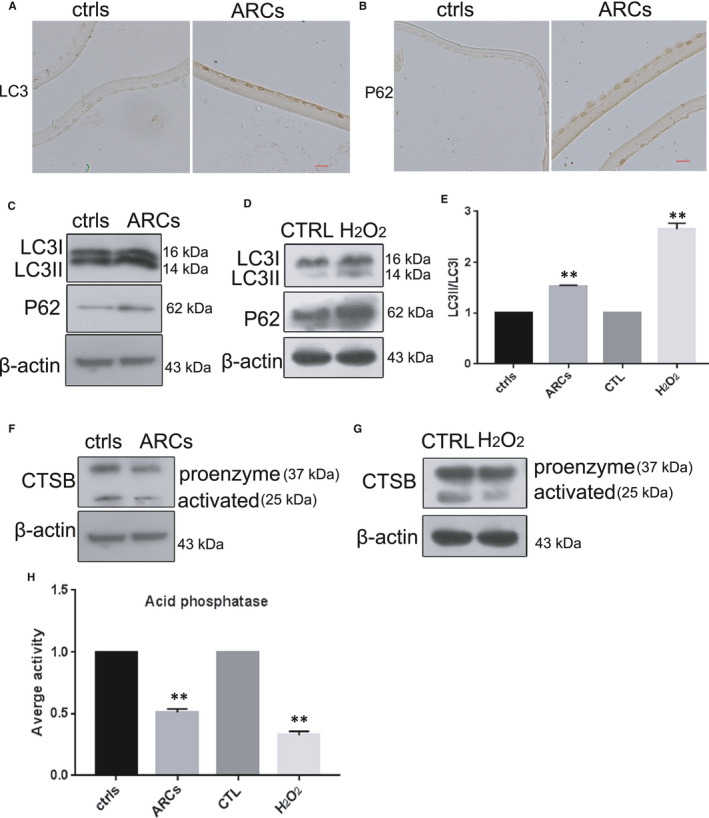
Impairment of autophagic flux and lysosomal dysfunction in the human lens epithelium of ARCs and in HLE‐B3 cells with oxidative stress‐induced senescence. (A). Representative images from IHC assays against LC3 in the controls (left) and ARCs (right). (B). Representative images from IHC assays against P62 in the controls (left) and ARCs (right). **(**C). Western blot analysis of LC3, P62 and β‐actin in the controls and ARCs. (D). Western blot analysis of LC3, P62 and β‐actin in untreated cells and the H_2_O_2_ (150 μM)‐treated cells. (E). Relative fold‐changes in the protein levels of LC3‐II/I as described in C and D. (F). Western blot analysis of CTSB and β‐actin in the controls and ARCs. The β‐actin was the same as that in C, as they were from the same sample. (G). Western blot analysis of CTSB and β‐actin in untreated cells and the H_2_O_2_ (150 μM)‐treated cells. The β‐actin was the same as that in D, as they were from the same sample. (H). Relative fold‐changes in the protein levels of CTSB as described in F and G. *, *p *< 0.05; **, *p *< 0.01 compared to the indicated sample. The bar represents 50 um

Several studies have shown that impairment of autophagic flux is a feature of cellular senescence.^27^ Thus, we examined whether autophagic flux was impaired in the lens epithelium of ARCs and in HLE‐B3 cells with oxidative stress‐induced senescence. As indicators of autophagy, the conversion of LC3‐I to LC3‐II and the expression of P62 were assessed using Western blotting. The results showed an increase in the LC3‐II/I ratio and P62 expression in the lens epithelium of ARCs compared to the controls (Figure 6C,E). In addition, the IHC results suggested that there was a significant difference in the LC3‐II/I ratio and expression of P62 between ARCs and the controls (Figure 6A,B). As shown in Figure 6D,E, there was dramatic accumulation of LC3‐II in senescent HLE‐B3 cells. In addition, the protein levels of LC3‐II and P62 were markedly increased in HLE‐B3 cells with oxidative stress‐induced senescence compared with normal HLE‐B3 cells. Surprisingly, the protein level of the activated form of cathepsin B (CTSB), which plays an essential role in lysosomal degradation of proteins, was obviously decreased in the lens epithelium of ARCs and H_2_O_2_‐induced HLE‐B3 cells (Figure 6F,G,H). These results suggest that senile HLE‐B3 cells had impaired autophagic flux and lysosomal dysfunction.

### 3.7 MET restored oxidative stress‐impaired autophagic flux via AMPK activation

**FIGURE 7:**
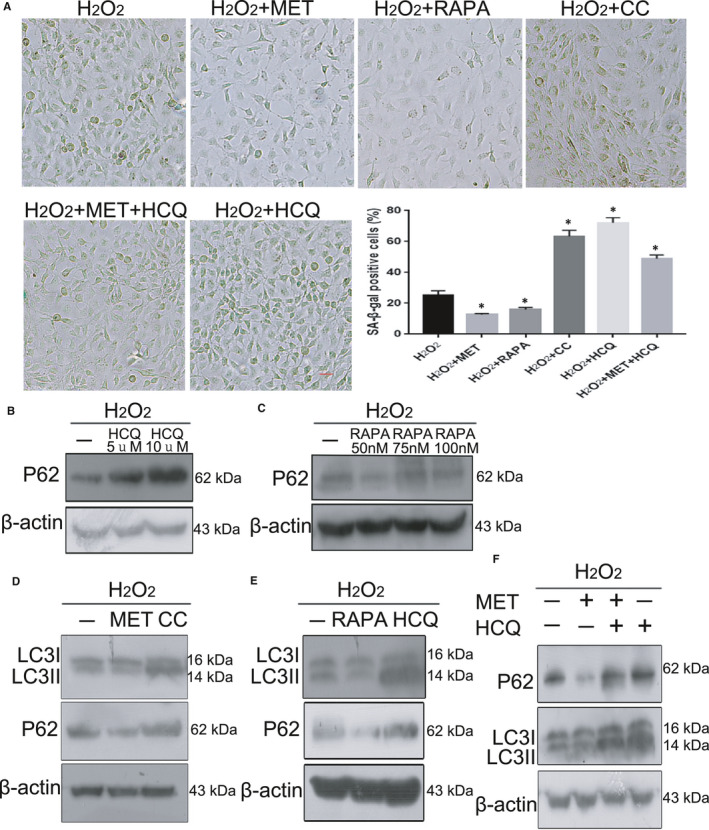
MET restored oxidative stress‐impaired autophagic flux via AMPK activation. (A). Representative images of SA‐β‐Gal staining of the cells and percentages of SA‐β‐Gal‐positive cells. (B). Western blot analysis of P62 and β‐actin in HLE‐B3 cells treated with HCQ at different concentrations. (C). Western blot analysis of P62 and β‐actin in HLE‐B3 cells treated with RAPA at different concentrations. (D). Western blot analysis of LC3, P62 and β‐actin in HLE‐B3 cells treated with H_2_O_2_ (150 μM)_,_ MET (2 mM) and CC (4 mM). (E). Western blot analysis of LC3, P62 and β‐actin in HLE‐B3 cells treated with H_2_O_2_ (150 [150 μM]), RAPA [50 nM] and HCQ [10 μM]). (F). Western blot analysis of LC3, p62 and β‐actin in HLE‐B3 cells treated with H_2_O_2_ (150 μM)_,_ MET (2 mM) and HCQ (10 μM). *p < 0.05; **p < 0.01 compared to the indicated sample. The bar represents 20 um

To further investigate the effect of MET on autophagic flux in our senescence system, we conducted a series of in vitro experiments. First, our results showed treatment with RAPA, an inducer of autophagy, significantly decreased the number of SA‐β‐gal‐positive cells to protect HLE‐B3 cells against H_2_O_2_‐induced impairment (Figure 7A). On the other hand, HCQ, a known autophagy inhibitor, conspicuously increased the number of SA‐β‐gal‐positive cells. MET weakened the negative effect of HCQ, resulting in a decrease in the number of SA‐β‐gal‐positive cells. Next, Western blot analysis showed that, similar to RAPA, MET notably reduced the H_2_O_2_‐induced protein levels of P62 and LC3‐II. In contrast, similar to HCQ, CC significantly suppressed the degradation of P62 and LC3‐II proteins (Figure 7B,C,D,E). Unsurprisingly, HCQ markedly increased the accumulation of P62 and LC3‐II proteins. Importantly, MET alleviated the HCQ‐induced inhibition of accumulation of P62 and LC3‐II proteins (Figure 7F). Our results suggest that MET restored oxidative stress‐impaired autophagic flux via AMPK activation.

### 3.8 MET restored the oxidative stress‐impaired autophagic flux associated with improvements in lysosomal function and mTOR inactivation

**FIGURE 8:**
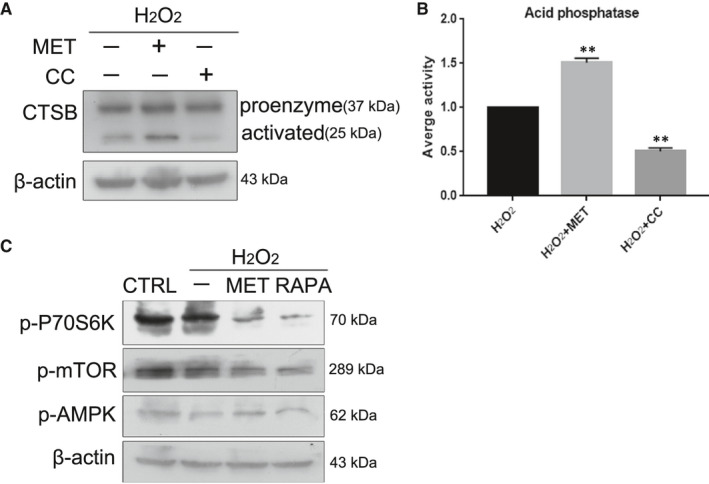
MET restored the oxidative stress‐impaired autophagic flux associated with improvements in lysosomal function and mTOR inactivation. (A). Western blot analysis of CTSB and β‐actin. (B). Relative fold‐changes in the protein levels of CTSB as described in A. (C). Western blot analysis of p‐P70S6K (Thr389), p‐MTOR (Ser2448), p‐AMPKα (Thr172) and β‐actin in HLE‐B3 cells treated with H_2_O_2_ (150 μM)_,_ MET (2 mM), RAPA (50 nM). **p < 0.01 compared to the indicated sample

As previously described, MET could partially prevent H_2_O_2_‐induced senescence in HLE‐B3 cells by improving autophagic flux. Additionally, the association between lysosomes and senescence has been extensively reported. It is generally acknowledged that mTOR signalling is involved in senescence. To determine the mechanism by which MET restored H_2_O_2_‐impaired autophagic flux in the context of lysosomal function, CTSB was used to evaluate the function of lysosomes via Western blotting. CTSB activation was evidently increased when AMPK was activated by MET and was obviously downregulated when AMPK was blocked by CC (Figure 8A,B). This result indicated that MET promoted the abundance of both the proenzyme and activated forms of CTSB, as well as the activity of lysosomal acid phosphatase. Then, we observed that the protein levels of phosphorylated mTOR (Ser2448) and phosphorylated p‐70S6K (Thr389) were distinctly suppressed in senescent HLE‐B3 cells. Thus, MET treatment played a robust role in inhibiting mTOR phosphorylation (Ser2448) and p‐70S6K phosphorylation (Thr389). Similar results were observed in HLE‐B3 cells treated with RAPA. Moreover, RAPA treatment downregulated the protein levels of phosphorylated AMPKα (Thr172), which indicated that the AMPK pathway was inactivated (Figure 8C). These results showed that MET restored the oxidative stress‐impaired autophagic flux that was associated with improved lysosomal function and mTOR inactivation.

## 4 DISCUSSION

Numerous scientific investigations have confirmed that MET can alleviate senescence by activating the AMPK pathway.^15^, ^26^, ^28^ In the present study, we provided convincing evidence that MET alleviated oxidative stress‐induced senescence stimulated via AMPK activation in vitro (Figure 5). Interestingly, we also discovered that MET restored the autophagic flux that was impaired by oxidative stress via AMPK activation in vitro (Figure 7), and this restoration was also related to the promotion of lysosomal function and mTOR inactivation (Figure 8). Notably, we detected AMPK inactivation and impaired autophagic flux in the lens epithelium of ARC (Figures 3,6). Our findings will be instructive for more intensive studies of MET‐mediated senescence inhibition. As an AMPK pathway activator, MET is expected to be an important target for new strategies against the formation and development of ARC.

Increasing evidence has indicated that oxidative damage is a predominant contributor to the pathogenesis of ARC.^29^, ^30^ Exogenous H_2_O_2_ has been widely used to induce cellular senescence. To simulate the ageing process in human lens epithelial cells to the maximum extent, long‐term low‐dose H_2_O_2_ treatment was used in lens epithelial cells. In this study, we showed that exogenous H_2_O_2_ could induce HLE‐B3 cell senescence (Figure 2). The ageing human lens epithelium of ARC is also characterized by common hallmarks of cellular senescence, including increased expression of P21 and P53 and increased SA‐β‐gal activity (Figure 1). In addition, the expression pattern of ageing genes in senescent HLE‐B3 cells and in the human lens epithelium of ARC is consistent (Figures 1,2). Successful establishment of human lens epithelial cell ageing was the basis for the success of the subsequent experiments.

It is widely accepted that MET stimulates AMPK, which is a key regulator of metabolic homeostasis in cells.^31^ Dysregulation of the AMPK pathway is a serious problem for cells and organisms. A series of studies focused on the link between AMPK and cellular senescence showed that AMPK activation plays a positive role in the anti‐senescence effect.^15^, ^24^, ^28^ In our study, under oxidative stress, which is generally acknowledged as one of the causes of cataracts, the anti‐senescence effect of the AMPK pathway was particularly prominent in HLE‐B3 cells (Figure 5). More interestingly, we observed that the AMPK pathway was activated in the human lens epithelium of ARC compared to the controls (Figure 3). These results provide meaningful evidence that the activation of AMPK may be an important target for delaying the occurrence of ARC. The AMPK pathway influences cellular senescence by modulating a complex network that includes P53, mTOR and FOXOs.^32^ On the basis of the above results, the molecular mechanisms linking AMPK activation to senescence prevention are worth further investigation.

Autophagy is a catabolic process to destroy and remove unnecessary or damaged components in cells and is regulated by conditions such as oxidative stress and starvation.^33^ The association between autophagy and senescence has been widely reported, and the role of MET in senescence inhibition is generally due to its effects on the induction of autophagy.^34^ There is a growing body of evidence, demonstrating that MET can directly prevent senescence by restoring autophagic flux.^35^ However, some scholars insist that MET can alleviate the senescence by promoting autophagy activity via AMPK activation.^36^ Our results strongly support the latter notion that MET significantly enhances autophagic flux by activating the AMPK pathway to delay oxidative stress‐induced senescence in HLE‐B3 cells (Figure 7). Our results show that oxidative stress damages the metabolism of lysosomes and leads to inhibition of P62 degradation in both senescent HLE‐B3 cells in vitro and the human lens epithelium of ARC (Figure 6B,C,D,E). Moreover, we discovered marked impairment of autophagic flux in the human lens epithelium of ARC (Figure 6A,B,C,E). In addition, we measured the expression of CTSB to explore the function of lysosomes in autophagosome degradation, which was associated with the late phase of autophagy (Figure 6F,G,H).

As previously mentioned, lysosomal function was noticeably damaged in both senescent HLE‐B3 cells and the human lens epithelium of ARC. Our results further confirmed that MET restored autophagic flux to promote lysosomal function (Figure 8A,B). MET enhanced the lysosomal degradation of autophagosomes, which is recognized as the late stage of autophagy. However, these data were different from previous results showing that MET could prevent oxidative stress‐induced senescence by restoring autophagic flux and mitochondrial functions in HLE‐B3 cells.^37^ Moreover, it is worth noting that the effect of MET on autophagic flux was similar to that of RAPA, which is also known as an mTOR inhibitor. Therefore, we believe that MET slowed oxidative stress‐induced senescence in association with mTOR inactivation (Figure 8C). In fact, consistent results have been detected by others.^38^ In addition, our results showed that the protein levels of p‐AMPK, p‐mTOR and p‐p70S6K were higher in the control group than in the other groups (Figure 8C). These findings suggest that the data presented here could explain the role of MET in delaying senescence under oxidative stress injury conditions. Whether MET can prevent normal cells from ageing still needs further study.

In conclusion, in our H_2_O_2_‐induced senescence model, our results suggested that the anti‐senescence effect of MET depended on activation of the AMPK pathway, which then activated autophagy. Furthermore, we also demonstrated that MET delays senescence by activating the AMPK pathway, improving lysosomal function and downregulating mTOR expression. Moreover, activation of the AMPK pathway is critical to the anti‐senescence effect of MET. Moreover, we found that senescence of the human lens epithelium in cataracts led to the inactivation of AMPK and inhibition of autophagy. Overall, these findings support MET as an emerging candidate for alleviating the formation and development of ARC and indicate a direction for our next experiments.

## CONFLICT OF INTEREST

The authors declare that they have no conflict of interest.

## AUTHOR CONTRIBUTIONS

**Mengmeng Chen:** Conceptualization (equal); Data curation (lead); Formal analysis (lead); Investigation (lead); Methodology (lead); Resources (equal); Software (lead); Supervision (lead); Validation (lead); Visualization (lead); Writing‐original draft (lead). **Chunmei Zhang:** Conceptualization (equal); Investigation (equal); Methodology (equal). **Nan Zhou:** Conceptualization (equal); Funding acquisition (equal); Project administration (equal). **Xu Wang:** Data curation (equal); Investigation (equal); Methodology (equal). **Dongmei Su:** Methodology (equal). **Yanhua Qi:** Conceptualization (lead); Funding acquisition (lead); Project administration (lead); Resources (lead); Writing‐review & editing (lead).

## Supporting information

Supplementary Material

## Data Availability

All data generated or used during the study are available from the corresponding author by request.
