# Supplementary material for: Metformin alleviates oxidative stress‐induced senescence of human lens epithelial cells via AMPK activation and autophagic flux restoration
Source: J Cell Mol Med. 2021 Jul 23;25(17):8376–89. doi: 10.1111/jcmm.16797 (PMC8419182; doi:10.1111/jcmm.16797)
Supplement: Supplementary file 1 — Supplementary Material [file JCMM-25-8376-s001.docx]

**Metformin Alleviates Oxidative Stress-induced Senescence of Human Lens Epithelial Cells via AMPK Activation and Autophagic Flux Restoration**

**Mengmeng Chen^1^, Chunmei Zhang^1^, Nan Zhou^1^, Xu Wang^2^, Dongmei Su^3^, Yanhua Qi^1^.**

**^1^**Department of Ophthalmology, the Second Affiliated Hospital of Harbin Medical University, Harbin, 150001, China.

**^2^** Department of Ophthalmology, Xixi Hospital of Hangzhou, Hangzhou, 310023, China.

**^3^**Department of Genetics, National Research Institute for Family Planning, Health Department, Beijing, 100000, China.

Correspondence to: Yanhua Qi; email: [qyh86605643@126.com](mailto:qyh86605643@126.com).

Table 1

Primer sequences in our experiment

Gene Primer Length of Gene ID

production

p21 F 5’-CGATGGAACTTCGACTTTGTCA-3’ 219bp [NM_000389.5](https://www.ncbi.nlm.nih.gov/nuccore/NM_000389.5)

R 5’-GCACAAGGGTACAAGACAGTG-3’

p16 F 5’-GAGCAGCATGGAGCCTTC-3’ 126bp [NM_000077.4](https://www.ncbi.nlm.nih.gov/nuccore/NM_000077.4)

R 5’-GGCCTCCGACCGTAACTATT-3’

IL-6 F 5’-ACTCACCTCTTCAGAACGAATTG-3’ 149bp [NM_000600.5](https://www.ncbi.nlm.nih.gov/nuccore/NM_000600.5)

R 5’-CCATCTTTGGAAGGTTCAGGTTG-3’

IL-8 F 5’-TTGGCAGCCTTCCTGATTTC-3’ 248bp [NM_000584.4](https://www.ncbi.nlm.nih.gov/nuccore/NM_000584.4)

R 5’-AACTTCTCCACAACCCTCTGCA-3’

FAS F 5’-GTGAGGGAAGCGGTTTACGA-3’ 193bp [NM_000043.6](https://www.ncbi.nlm.nih.gov/nuccore/NM_000043.6)

R 5’-AGATGCCCAGCATGGTTGTT-3’

β-actin F 5′- TCGTGCGTGACATTAAGGAG-3′ 303bp [NM_001101.5](https://www.ncbi.nlm.nih.gov/nuccore/NM_001101.5)

R 5′-ATGCCAGGGTACATGGTGGT′-3

**Figure 1**


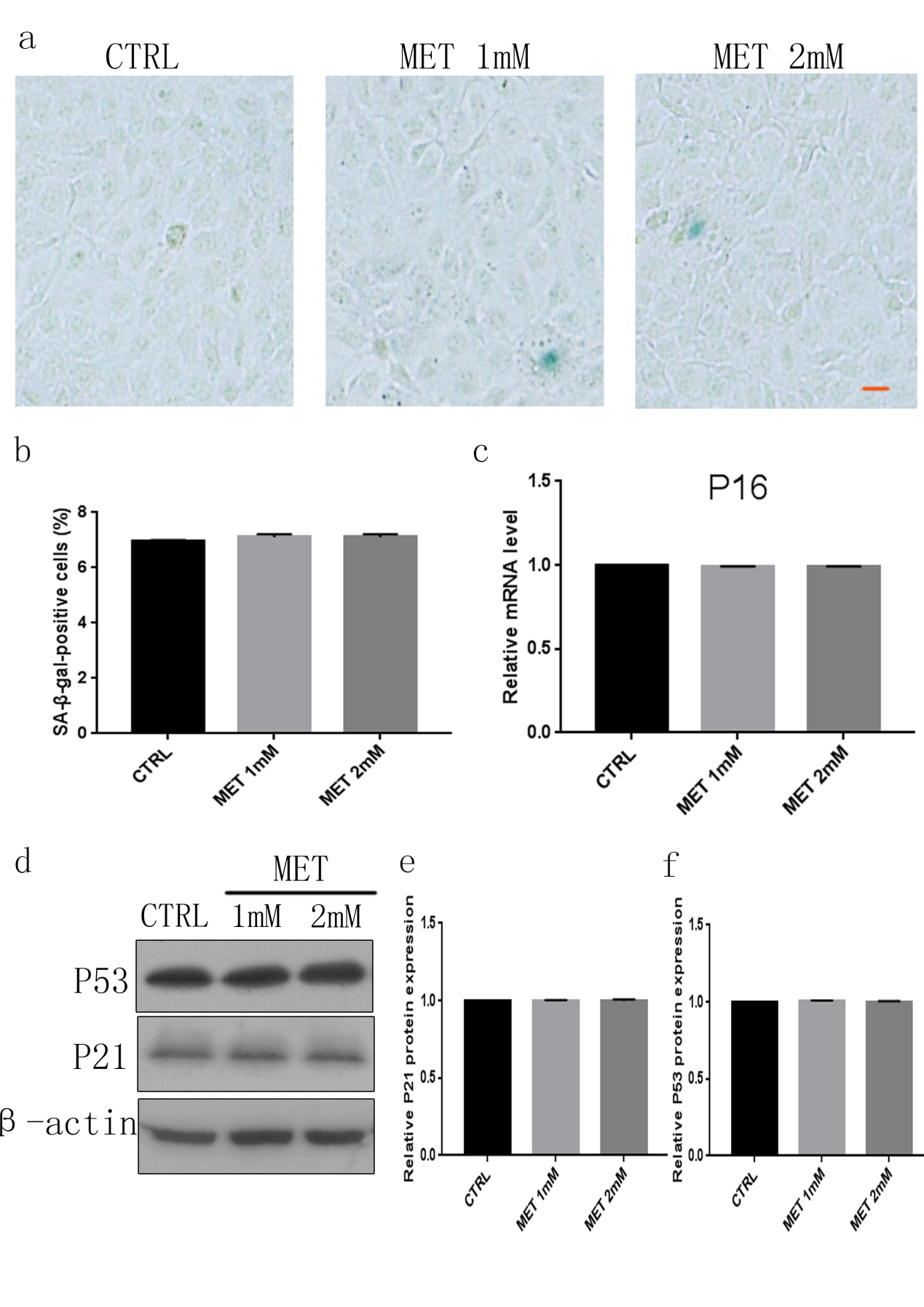


**Figure 1 Identification of senescent HLE-B3 cells in CTRL and MET only groups.** HLE-B3 cells were treated with MET only at different concentrations for 7 days. CTRL means untreated cells. **(a).** Representative images of SA-β-Gal staining of the cells. **(b).** Percentages of SA-β-Gal positive cells. **(c).** Relative fold-changes in the mRNA levels of the genes encoding P16 as determined by qRT-PCR. **(d).** Western blot analysis of P53, P21 and β-actin in HLE-B3 cells treated with MET only at different concentrations for 7 days. **(e).** Relative fold-changes in the protein levels of P21 as described in **d. (f)** Relative fold-changes in the protein levels of P53 as described in **d.** Data were shown as mean ± SD and are representative of 3 independent experiments. p>0.05 compared to the control (CTRL). The bar represents 20ｕm.
